# Subcellular localization and interactions among rubber particle proteins from *Hevea brasiliensis*

**DOI:** 10.1093/jxb/erx331

**Published:** 2017-09-25

**Authors:** Daniel Brown, Mistianne Feeney, Mathin Ahmadi, Chiara Lonoce, Roslinda Sajari, Alessandra Di Cola, Lorenzo Frigerio

**Affiliations:** 1 School of Life Sciences, University of Warwick, Coventry, UK; 2 Tun Abdul Razak Research Centre, Brickendonbury, Hertford, UK; 3 Malaysian Rubber Board, Experiment Station, Sungai Buloh, Selangor DE, Malaysia

**Keywords:** Biogenesis, endoplasmic reticulum, *Hevea*, latex, particle, plant, rubber

## Abstract

Natural rubber (polyisoprene) from the rubber tree *Hevea brasiliensis* is synthesized by specialized cells called laticifers. It is not clear how rubber particles arise, although one hypothesis is that they derive from the endoplasmic reticulum (ER) membrane. Here we cloned the genes encoding four key proteins found in association with rubber particles and studied their intracellular localization by transient expression in *Nicotiana benthamiana* leaves. We show that, while the *cis*-prenyltransferase (CPT), responsible for the synthesis of long polyisoprene chains, is a soluble, cytosolic protein, other rubber particle proteins such as rubber elongation factor (REF), small rubber particle protein (SRPP) and Hevea rubber transferase 1-REF bridging protein (HRBP) are associated with the endoplasmic reticulum (ER). We also show that SRPP can recruit CPT to the ER and that interaction of CPT with HRBP leads to both proteins relocating to the plasma membrane. We discuss these results in the context of the biogenesis of rubber particles.

## Teaser

The major proteins of rubber particles from *Hevea brasiliensis* associate with the endoplasmic reticulum membrane and can interact with the key cytosolic biosynthetic enzyme *cis*-prenyltransferase.

## Introduction

Natural rubber (NR) is a globally essential, industrial raw material used in the manufacture of a vast array of products, ranging from aircraft and vehicle tyres to medical apparel and devices. Despite numerous attempts to find alternative plants for the production of NR, the rubber tree *Hevea brasiliensis* is still, irreplaceably, the main commercial source for the NR industry worldwide.

NR is a polymer of *cis*-polyisoprene. Rubber biosynthesis takes place in specialized phloem cells known as laticifers and, more specifically, on the surface of the most abundant laticifer subcellular compartments, the rubber particles (RPs) (Archer *et al.*, 1963).

To date, the most abundant proteins found to be involved in rubber biosynthesis are isopentenylpyrophosphate (IPP) polymerizing enzyme, *cis*-prenyltransferase (CPT); rubber elongation factor (REF) and small rubber particle protein (SRPP) (Light *et al.*, 1989; Oh *et al.*, 1999; Asawatreratanakul *et al.*, 2003). A proteomic study of large rubber particles (LRPs) and small rubber particles (SRPs) isolated from *Hevea* latex confirms rubber particle localization and distribution of these proteins: REF and SRPP are found to be the most abundant RP proteins, with REF (14.7 kDa isoform) being equally expressed on both types of RPs, but a heavier REF isoform (19.6 kDa) being exclusively found in LRPs, and SRPP only present in SRPs (Xiang *et al.*, 2012). Both REF and SRPP were originally identified as Hevb1 and Hevb3, respectively, and known to be two of the main allergens responsible for latex allergy. REF was demonstrated to affect IPP incorporation in isolated rubber particles: disruption of REF resulted in a block of IPP polymerization (Dennis and Light, 1989). SRPP was found associated to SRPs and its role in rubber biosynthesis was demonstrated with recombinant SRPP increasing IPP incorporation in rubber biosynthesis assays *in vitro* (Oh *et al.*, 1999). In addition, recombinant SRPP was shown to coat model monolayer membranes, whereas REF appears to be more tightly embedded into the membrane (Berthelot *et al.*, 2014).

The recent publication of *Hevea brasiliensis* annotated genomes (Lau *et al.*, 2016; Tang *et al.*, 2016) revealed the complexity of the gene families for RP-associated biosynthetic enzymes: 11 CPT, 8 REF and 10 SRPP isoforms were identified (Tang *et al.*, 2016). Although from tissue-specific transcriptomic data it would be possible to restrict the analysis to the latex-specific isoforms, more investigation is needed to unravel the nature of the variants and their involvement in rubber biosynthesis.

**Fig. 1.**
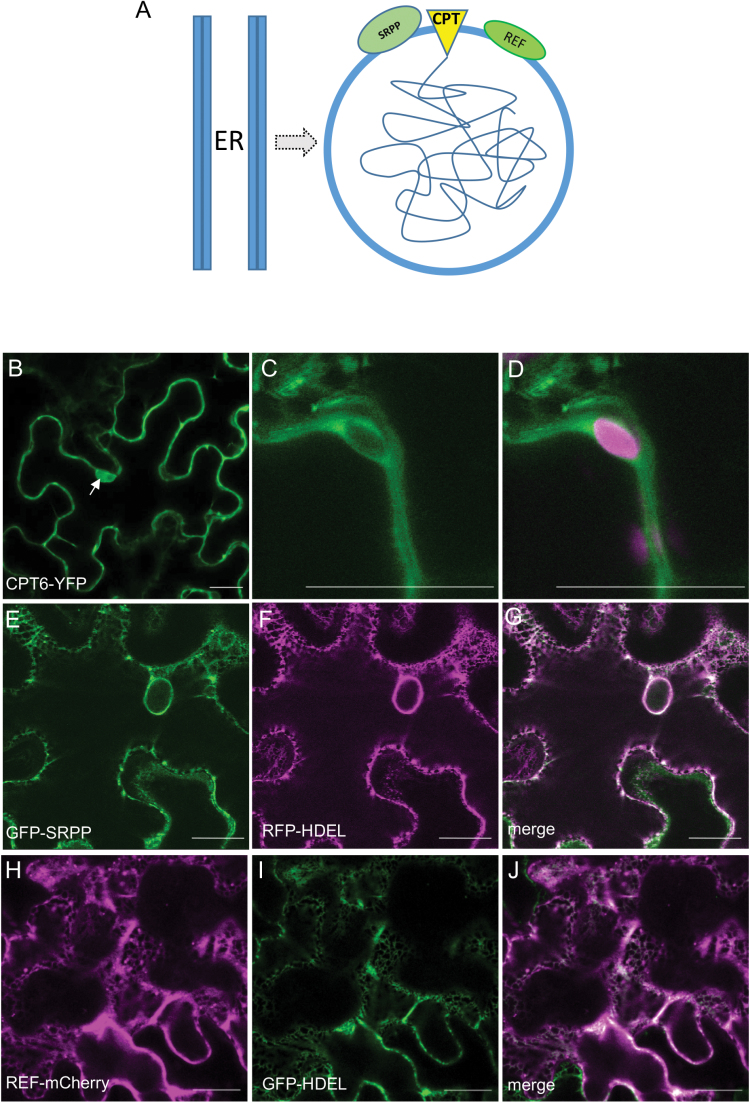
*Hevea* proteins SRPP and REF are localized to the endoplasmic reticulum and CPT6 is localized to the cytoplasm. (A) Current model of rubber particle biogenesis. Rubber particles possibly originate from the endoplasmic reticulum (ER). The core of polyisoprene (tangled line) is surrounded by a monolayer membrane. The *cis*-prenyltransferase (CPT) and the accessory proteins REF and SRPP are all assumed to be associated with the ER membrane. (B–J) *N. benthamiana* leaves were agroinfiltrated with the indicated constructs and leaf sectors were imaged after 3 d. (B–D) CPT6-YFP shown in green (A–C) and chlorophyll autofluorescence shown in red (C). (E–G) GFP-SRPP shown in green (D) co-expressed with RFP-HDEL shown in red (E). (H–J) REF-mCherry shown in red (E) co-expressed with GFP-HDEL shown in green (F). The arrow indicates CPT6-YFP signal in the nucleoplasm. Scale bars: 20 μm.

Large and small RPs are surrounded by a lipid monolayer membrane, as schematized in Fig. 1A. The lipid composition of the monolayer suggests that RPs may originate from the membrane of the endoplasmic reticulum (ER) (Cornish *et al.*, 1999; Chrispeels and Herman, 2000). Rubber particles have been described as lipid droplets storing rubber rather than triacylglycerols, as in the better characterized lipid bodies (Laibach *et al.*, 2015). Therefore, current models for RP formation are based on the analogy with the biogenesis of oil bodies (Herman, 2008). This implies that all key proteins involved in rubber biosynthesis are ER membrane-associated proteins (Fig. 1A). The fact that REF is also found on the membrane of oil bodies (Horn *et al.*, 2013) seems to support this hypothesis. In order to test this hypothesis, we have investigated the subcellular localization and the patterns of interaction of all the factors that are known to be involved in rubber biosynthesis and associated to RPs, by transient expression in *Nicotiana benthamiana* leaves. We chose a non-latex plant background as this would be the ideal blank canvas for studying the initial events of rubber particle biogenesis, highlighting the subcellular precursor compartment that is at the origin of their formation. Here we focused on one isoform of *cis*-prenyltransferase (CPT6) and on selected isoforms of the other major RP proteins, SRPP2 (Oh *et al.*, 1999) and REF1 (Light *et al.*, 1989) as identified by proteomic analysis (Dai *et al.*, 2013). Tang *et al.* (2016) identified 11 isoforms of CPT. Of these only CPT6, 7 and 8 have detectable expression in latex. CPT6 (formerly named HRT2) was initially characterized as being the most active CPT, based on recombinant protein activity assays in the presence of RPs (Asawatreratanakul *et al.*, 2003). We cloned this isoform from the draft genome of the *Hevea brasiliensis* clone RRIM928 (Malaysian Rubber Board, unpublished). Amongst the eight REFs and 10 SRPPs, REF1 and SRPP2 have high expression levels in latex and were initially found to be associated to RPs (Dai *et al.*, 2013).

We also identified the *Hevea* homologue of the *Taraxacum brevicorniculatum* rubber transferase activator (TbRTA), recently renamed Hevea rubber transferase 1 (HRT1)-REF bridging protein (HRBP; Yamashita *et al.*, 2016)—a newly described protein that is a homologue of the mammalian Nogo B receptor (NgBR) and is directly involved in the regulation of CPT. Knockdown of TbRTA resulted in the block of rubber biosynthesis (Epping *et al.*, 2015). Our results indicate that, while CPT6 is a cytosolic protein, SRPP, REF and HRBP localize to the ER. Both SRPP and HRBP can alter the localization of CPT6. These data provide an initial framework for understanding the interactions between rubber particle proteins.

## Materials and methods

### Constructs

A complete list of oligonucleotide primers used for cloning the coding sequences used in this study is shown in Supplementary Table S1 at *JXB* online. Coding sequences for all proteins used in this work were cloned into either binary vector pGREEN0029 (Hellens *et al.*, 2000) or into Gateway destination vectors. A table of all constructs is shown in Supplementary Table S2.

### Identification and cloning of HRBP

*cis*-Prenyltransferase-like (CPTL) protein sequences for Arabidopsis LEAF WILTING 1 (LEW1; Zhang *et al.*, 2008) and *T. brevicorniculatum* TbRTA (Epping *et al.*, 2015) were used initially as queries against the public database. There were no BLAST hits for a *Hevea* protein. The CPTL sequences were then used as a query for a pBLAST against Tun Abdul Razak Research Centre (TARRC)’s *Hevea* predicted protein database (unpublished). A protein from the database with the ID tag ‘HEVBR187338_AB_0629490’ displayed a sequence identity of roughly 50% and was named temporarily HB50. The genome scaffold, >scaffold_161569.fa_seg, that contained the nucleotide sequence for HB50 was found using HB50 as a protein query against the nucleotide genome sequence. The open reading frame (ORF) of >scaffold_161569.fa_seg were annotated by using NBCI BLAST to compare the sequence against the public database. This revealed several close hits to predicted Nogo B receptor-like proteins and dedol-PP synthases from other plant species. It was also apparent from NCBI that the HB50 gene was split into three ORFs with exons in between, and that the 5′ region of the gene was missing from the scaffold, in a region of unknown sequence. Based upon the truncated version of HB50 in the *Hevea* scaffold sequence, primers were designed to amplify and sequence the gene from genomic DNA. This sequence was then used as the basis for further primer design, in order to sequence back into the unknown region, using genomic DNA as a template. Primers were designed to amplify the entire coding sequence from cDNA. The gene was named *HevNogo*, then renamed *HRBP* after the same sequence was identified by Yamashita *et al.* (2016).

### Transient expression

Liquid cultures of *Agrobacterium tumefaciens* strains GV3101 or C58 containing the indicated constructs were infiltrated into the abaxial side of 4-week-old of soil-grown *N. benthamiana* leaves as described (Sparkes *et al.*, 2006). Infiltrated leaves were imaged after 3 d or longer, as indicated. For staggered infiltration experiments, leaf sectors were initially infiltrated with the first construct. After 24 h, the same sectors were re-infiltrated with the second construct. Sectors were imaged 4 d after the first infiltration.

### Confocal microscopy

Leaf samples approximately 0.25 cm^2^ were excised from the plant and mounted on a microscope slide in water and imaged with either a Leica TCS SP5 or a Zeiss LSM 880 confocal microscope, using either a ×40 or ×63 oil objective lens. In some experiments leaf sectors were incubated with 5 µg ml^−1^ brefeldin A (BFA) for 1 h prior to washing in water and mounting. FM4-64 (Invitrogen) was used in some experiments to stain the plasma membrane of leaf cells. Leaf samples were incubated in 8 µM FM4-64 for 5 min and washed three times with water. Cyan fluorescent protein (CFP) was excited at 405 nm and detected in the 470–485 nm range; enhanced green fluorescent protein (eGFP) was excited at 488 nm and detected in the 495–520 nm range. Enhanced yellow fluorescent protein (eYFP) was excited at 514 nm and detected in the 525–550 nm range. mCherry was excited at 561 nm and detected in the 571–638 nm range. FM4-64 was excited at 514 nm and detected in the 616–645 nm range. Simultaneous detection of YFP and red fluorescent protein (RFP) was performed by sequential scanning according to the manufacturer’s instructions. The confocal microscope settings were kept constant throughout experiments. Image processing was done via LAS AF Lite or Zeiss Zen Blue edition, depending on the microscope used.

### Co-immunoprecipitation

*N. benthamiana* leaves were agroinfiltrated with the indicated pairs of constructs. After 3 d, infiltrated leaf sections were homogenized in homogenization buffer (150 mM Tris–HCl pH 7.5, 150 mM NaCl, 1.5% (v/v) Triton X-100), supplemented immediately before use with ‘Complete’ protease inhibitor cocktail (Roche Diagnostics, Burgess Hill, UK) and subjected to immunoprecipitation with GFPTrap or RFPTrap beads (Chromotek), following the manufacturer’s instructions. Beads equilibrated in wash buffer (10 mM Tris–HCl, pH 7.5, 150 mM NaCl, 0.5 mM EDTA) were added to the homogenates and the mixture tumbled for 2 h at 4 °C. The mixture was centrifuged at 376 *g*, 2 min, 4 °C, and a sample of supernatant was taken for analysis and the rest discarded. The beads were subsequently washed three times in wash buffer, resuspended in SDS-PAGE sample buffer and incubated at 37 °C for 10 min before loading. Immunoselected polypeptides were resolved by SDS-PAGE, transferred to polyvinylidene fluoride filters and subjected to immunoblotting with either anti-GFP (rat monoclonal) or anti-RFP (mouse monoclonal) antibodies (Chromotek).

## Results and discussion

### CPT6 is a cytosolic protein

In order to analyse its subcellular localization, CPT6 was tagged with YFP at either the N- or the C-terminus. 35S:CPT6-YFP and 35S:YFP-CPT6 were transformed into *A. tumefaciens* C58, infiltrated into the abaxial side of *N. benthamiana* leaves and imaged 3 d post-infiltration (dpi) (Fig. 1B–D). CPT6-YFP localization appeared to be cytosolic, and its fluorescence was detected in the space surrounding chloroplasts (Fig 1C, D). The predicted CPT6-YFP fusion protein size is 59 kDa, which is below the 60 kDa threshold for free diffusion into the nucleoplasm. This was confirmed by a strong signal in the nucleoplasm (Fig. 1B, arrow). This pattern is typical of a cytosolic protein, which confirms cytosolic localization of CPT6. The position of the fluorescent tag had no effect on the cellular localization of CPT6, with both CPT6-YFP and YFP-CPT6 configurations localizing to the cytoplasm (see Supplementary Fig. S1A, B). The CPT6-YFP signal was detectable throughout a 2-day observation window, indicating that CPT6 is a relatively stable protein. This observation is at odds with the data by Yamashita *et al.* (2016), which suggest that HRT1 and HRT2 (CPT7 and CPT6, respectively) are unstable proteins that need to be stabilized by interaction partners in order to be detectable.

### GFP-SRPP and REF-mCherry localize to the ER

We generated fluorescent protein fusions to SRPP and REF and expressed them in *N. benthamiana* (Fig. 1E–G and H–J, respectively). Both protein fusions displayed a clear ER pattern of localization, as indicated by the co-localization of GFP-SRPP with the ER luminal marker RFP-HDEL (Fig. 1E–G) and of REF-mCherry with GFP-HDEL (Fig. 1H–J). These results are in agreement with previously described localizations for these proteins (Yamashita *et al.*, 2016). Interestingly, neither REF nor SRPP is predicted to carry a signal peptide or contain any transmembrane domains according to the SignalP (Petersen *et al.*, 2011) and TOPCONS (Bernsel *et al.*, 2009) prediction algorithms, respectively.

### SRPP can recruit CPT6 to the ER

**Fig. 2.**
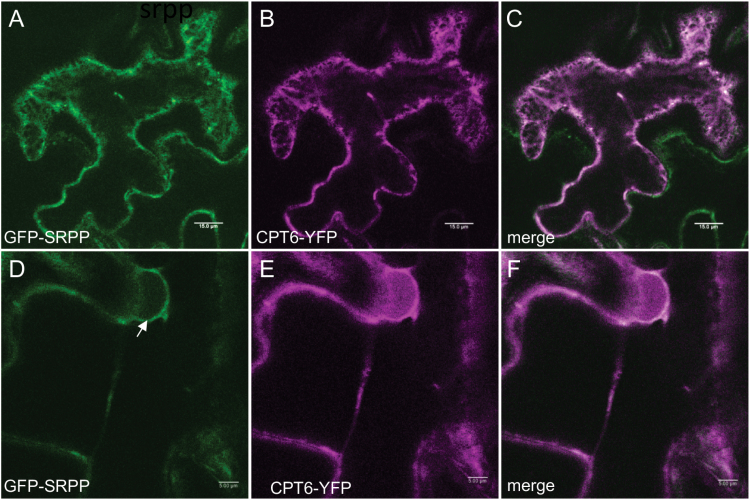
Co-expression of SRPP redirects CPT6 to the endoplasmic reticulum. *N. benthamiana* leaves were agroinfiltrated with both CPT6-YFP (red) and GFP-SRPP (green) and imaged after 3 d. The arrow points to the nuclear envelope. Scale bars: 15 μm (A–C) and 5 μm (D–F).

When we co-expressed CPT6-YFP with REF-mCherry, the fluorescence signals did not co-localize, with CPT6-YFP remaining cytosolic/nucleoplamic and REF-mCherry remaining ER associated (see Supplementary Fig. S2G–I). When, however, we co-infiltrated *N. benthamiana* leaves with the CPT6-YFP and GFP-SRPP constructs, we observed that the signals co-localized, with CPT6-YFP now also labelling the ER network (Fig. 2A–C) and the nuclear envelope (Fig. 2D–F). The same result was obtained by co-expressing GFP-SRPP and YFP-CPT6 (see Supplementary Fig. S3G–I). This indicates that SRPP co-expression can redirect CPT6 to the ER. A previous study from Berthelot *et al.* (2014) showed that REF and SRPP interact differently with RP membranes, with REF binding tighter that SRPP. Regardless of the high degree of homology between SRPP and REF (Berthelot *et al.*, 2014), our data show that CPT6 is recruited to the ER from the cytosol when in the presence of SRPP, but remains in the cytosol when REF is co-expressed. This observation is in agreement with Berthelot *et al.* (2014), suggesting that a more peripheral association of SRPP to the ER results in the protein being available for interaction with cytosolic CPT6, while a more tightly ER-embedded REF is not (Berthelot *et al.*, 2014).

### HRBP is an ER protein and it changes the localization of CPT6

**Fig. 3.**
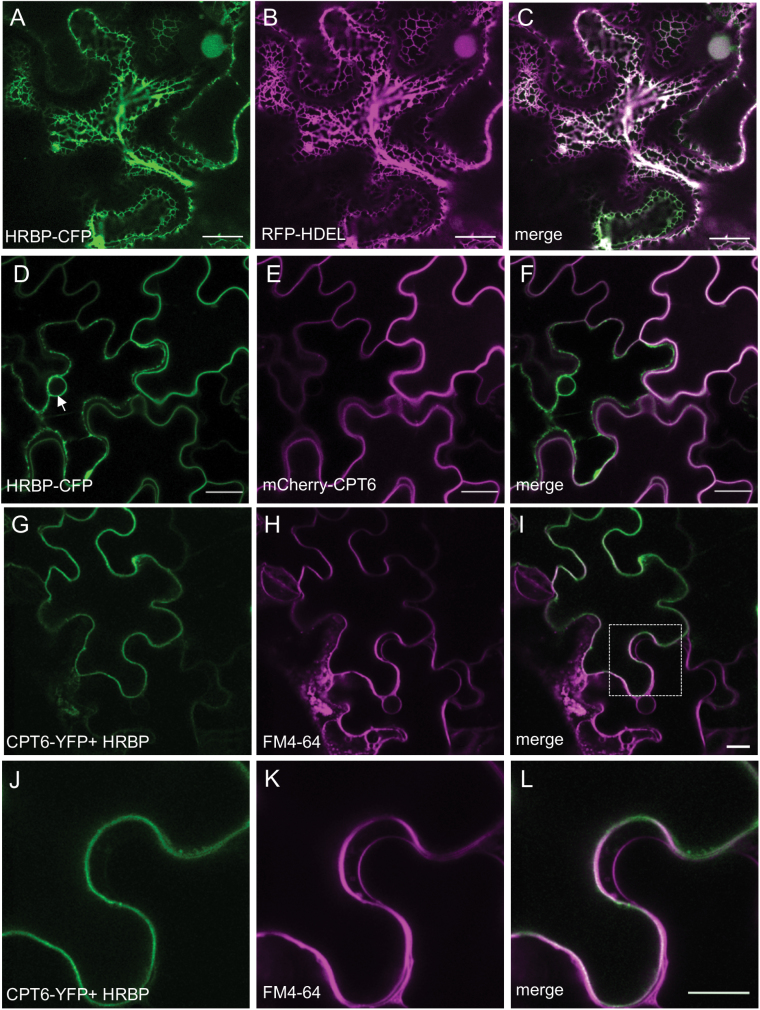
Co-expression of HRBP and CPT6 relocates both proteins to the plasma membrane. *N. benthamiana* leaves were agroinfiltrated with the indicated constructs. (A–C) HRBP-CFP (green) co-localizes with RFP-HDEL (red) on the ER. (D–F) HRBP-CFP signal (green) is observed on the ER and co-localizes with mCherry-CPT6 (red) on the plasma membrane. (G–I) Leaves co-infiltrated with CPT6-YFP (green) and untagged HRBP (G) were stained with FM4-64 in red (H). CPT6-YFP co-localizes with FM4-64 on the plasma membrane (I). (J–L) Magnified image of the area identified by the square in (I). Scale bars: 20 μm.

We identified and cloned the *Hevea brasiliensis* homologue of TbRTA (Epping *et al.*, 2015) in the genome of RRIM928. This protein was also recently reported by Yamashita *et al.* (2016) and named HRBP (HRT1-REF bridging protein). HRBP was tagged with CFP at either the C- or N-terminus. In *N. benthamiana* leaf epidermal cells, HRBP localized to the ER as indicated by co-expression with RFP-HDEL (Fig. 3A–C).

When we co-expressed HRBP-CFP with CPT6-mCherry, however, both proteins were detected quite clearly at a continuous structure at the edge of cells, which is likely to be the plasma membrane (Fig. 3D–F). Co-expression of the two proteins was necessary for plasma membrane localization of HRBP: within a field of agroinfiltrated cells, a few cells only expressing HRBP-CFP were still presenting an ER labelling pattern (Fig. 3D, arrow) whereas cells expressing both proteins showed a plasma membrane-like pattern (Fig. 3F).

To confirm that this structure was indeed the plasma membrane, and that this observation did not result from mis-sorting of the proteins due to fluorescent protein tag interference, we co-expressed CPT6-YFP with untagged HRBP (Fig. 3G–L). We observed the same pattern of CPT6-YFP localization as seen in the presence of HRBP-CFP. Incubation with the styryl dye FM4-64 confirmed that CPT6 (presumably in association with HRBP) is indeed at the plasma membrane (Fig. 3J–L). This result is intriguing, as it appears that the ER protein HRBP can both lose its ER localization and carry CPT6 to the plasma membrane upon co-expression.

**Fig. 4.**
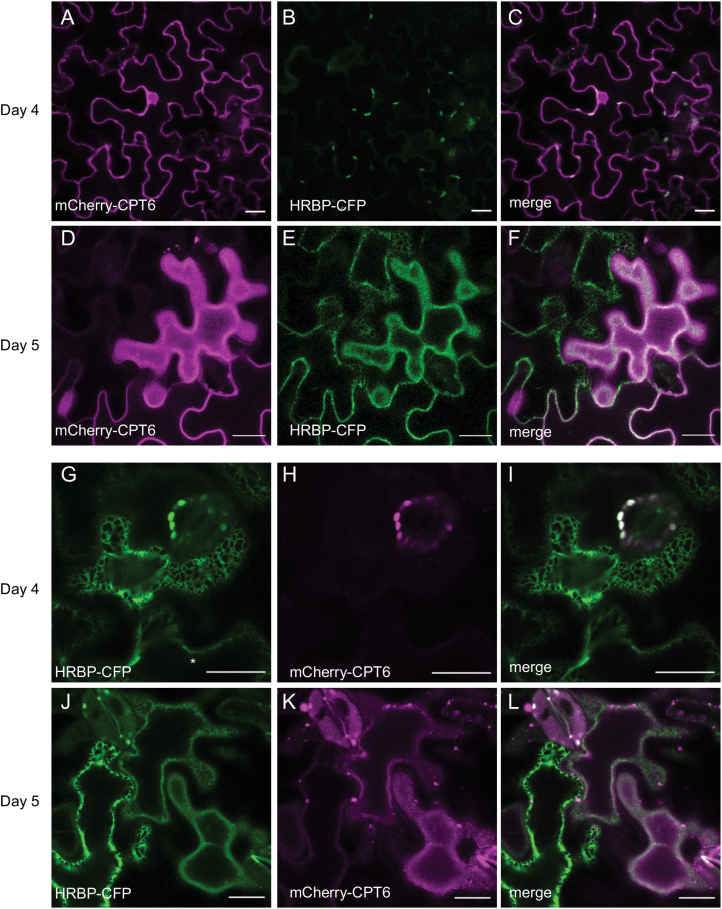
Time-dependent relocation of CPT6 and HRBP upon co-expression. Staggered co-infiltration of *N. benthamiana* leaves with mCherry-CPT6 (red) and HRBP-CFP (green). (A–L) Infiltration of mCherry-CPT6 followed 24 h later by HRBP-CFP (A–F) or by HRBP-CFP followed 24 h later by mCherry-CPT6 (G–L). Leaf sectors were imaged 4 d (A–C, G–I) or 5 d (D–F, J–L) after the first infiltration. Note that the construct infiltrated a day later is not yet visible at day 4 (B, H). Scale bars: 20 μm.

In order to test whether a causal link exists between HRBP-CFP and mCherry-CPT6 co-expression, with consequent co-localization and plasma membrane trafficking, we performed staggered co-infiltration experiments where mCherry-CPT6 was expressed 1 d earlier than HRBP-CFP, and a reciprocal experiment where HRBP was expressed 1 d earlier. This allowed us to observe the localization of the first protein before the second protein becomes expressed (Tolley *et al.*, 2008). In the former experiment, mCherry-CPT6, which initially appeared in the cytosol (Fig. 4A–C) relocated to the plasma membrane upon the onset of expression of HRBP-CFP (Fig. 4D–F). Likewise, in the reciprocal experiment, HRBP-CFP, which initially was entirely localized to the ER (Fig. 4G–I), gradually appeared to label the plasma membrane upon co-expression of mCherry-CPT6 (Fig. 4J–K).

**Fig. 5.**
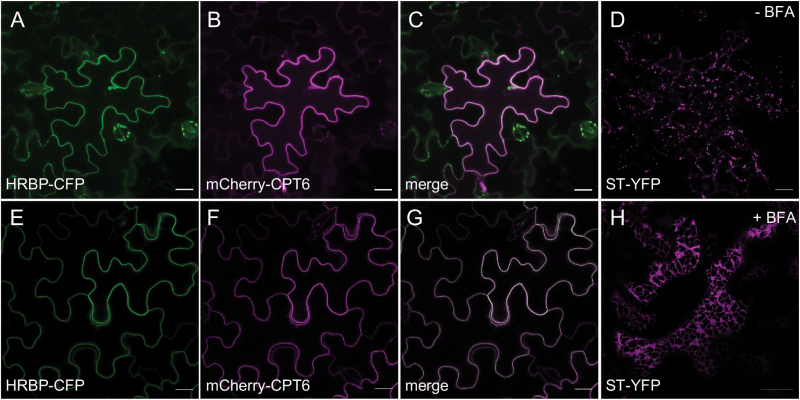
Plasma membrane trafficking of CPT6 and HRBP is insensitive to brefeldin A (BFA) treatment. *N. benthamiana* leaves were co-infiltrated with HRBP-CFP (green), mCherry-CPT6 (red) and the Golgi marker ST-YFP (magenta). Three days after infiltration, leaf sectors were imaged (A–D) and compared with leaf sectors incubated with 5 μg ml^−1^ BFA for 1 h (E–H). Scale bars: 20 μm.

As Yamashita *et al.* (2016) showed that HRT1 (CPT7) and HRBP localize to the Golgi complex when co-expressed, we tested whether the HRBP–CPT6 complex also travels through the Golgi on its way to the plasma membrane. We therefore co-expressed both proteins in *N. benthamiana* leaves and treated cells with BFA, which in *Nicotiana* leaves inhibits Golgi-mediated anterograde trafficking (Robinson *et al.*, 2008) (Fig. 5). BFA did not inhibit or reduce the appearance of the two proteins at the plasma membrane (Fig. 5, compare panels A–C with E–G), while it was able to relocate the Golgi marker ST-YFP to the ER (Fig. 5, compare panels D and H). Thus, it appears that trafficking of HRBP-CFP and mCherry-CPT6 to the plasma membrane is insensitive to BFA treatment and therefore unlikely to involve the Golgi complex.

### HRBP relies on an N-terminal domain for ER localization

**Fig. 6.**
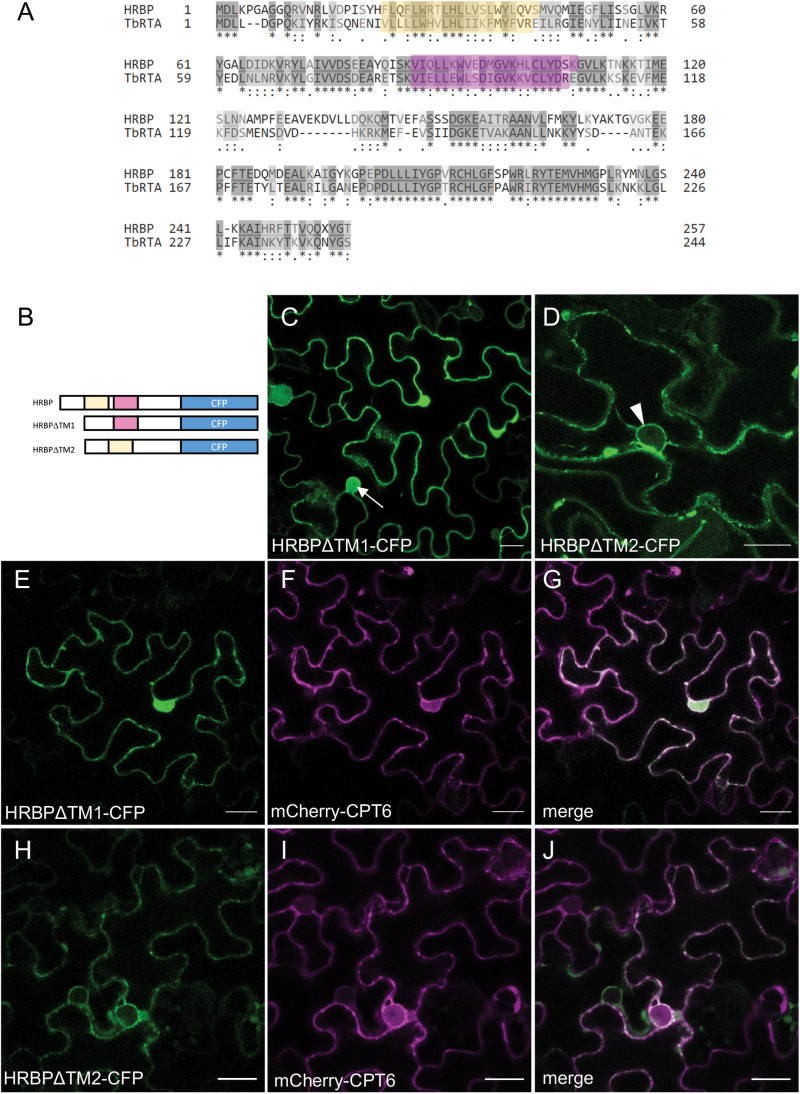
Deletions of putative transmembrane domain regions affect the location of HRBP. (A) Pairwise alignment of the HRBP and TbRTA protein sequences. The putative transmembrane domains, based on the topology of TbRTA, are highlighted in yellow and magenta. (B) Schematic representation of the full-length HRBP and HRBP mutants lacking putative transmembrane regions. (C–J) *N. benthamiana* leaves were co-infiltrated with HRBP∆TM1-CFP or HRBP∆TM2-CFP (green) and mCherry-CPT6 (red) and imaged after 3 d. Arrow in (C) points to HRBP∆TM1-CFP signal in the nucleoplasm. Arrowhead in (D) points to HRBP∆TM2-CFP signal in the nuclear envelope. Scale bars: 20 μm.

HRBP is an ER-associated protein. We used the TOPCONS prediction algorithm (Bernsel *et al.*, 2009; Tsirigos *et al.*, 2015) to search for putative transmembrane domains (see Supplementary Fig. S4). While there were no consensus transmembrane regions predicted by TOPCONS, a combination of the prediction algorithm and comparison with other known Nogo B receptor (NgBR) orthologues, in particular, alignment with TbRTA (Epping *et al.*, 2015), indicated that two potential transmembrane regions (TM1 and TM2) may be present in the sequence of HRBP (Fig. 6A). Plant NgBR orthologues Arabidopsis LEW1 (Zhang *et al.*, 2008) and *T. brevicorniculatum* TbRTA both have strong predictions for the presence of two transmembrane domains, whereas the confidence for *Hevea* HRBP transmembrane domains is low. Also, for the human NgBR, TOPCONS predicts with low confidence transmembrane domains with a similar result to HRBP. NgBR, however, has experimentally been shown to have multiple transmembrane domains that are in a similar position to LEW1 and TbRTA (Harrison *et al.*, 2011). Therefore, based on this combination of sequence analysis and experimental evidence for HRBP orthologues, we hypothesized the existence of two putative transmembrane regions for HRBP. Deletion analysis was performed at each of the putative TM regions, with subcellular localization analysed by confocal microscopy. Deletion of TM1 abolished ER localization, with the truncated protein displaying a typically cytosolic pattern (note signal in the nucleoplasm in Fig. 6C). Deletion of TM2, however, did not change the ER localization of the mutant protein (note the nuclear envelope labelling in Fig. 6D). When HRBPΔTM1-CFP was co-expressed with mCherry-CPT6, both proteins localized to the cytosol (Fig. 6E–G). When HRBPΔTM2-CFP and mCherry-CPT6 were co-expressed, both proteins localized to the ER, as indicated by the labelling of the nuclear envelope (Fig. 6H–J). This indicates that TM1 is necessary for HRBP-CFP localization to the ER. It also suggests that the second putative transmembrane region may be involved in the relocation of the HRBP–CPT6 complex from the ER to the plasma membrane, as deletion of this region abolishes plasma membrane (PM) localization but it still allows for HRBP to recruit mCherry-CPT6 to the ER.

### Interactions between rubber particle proteins

**Fig. 7.**
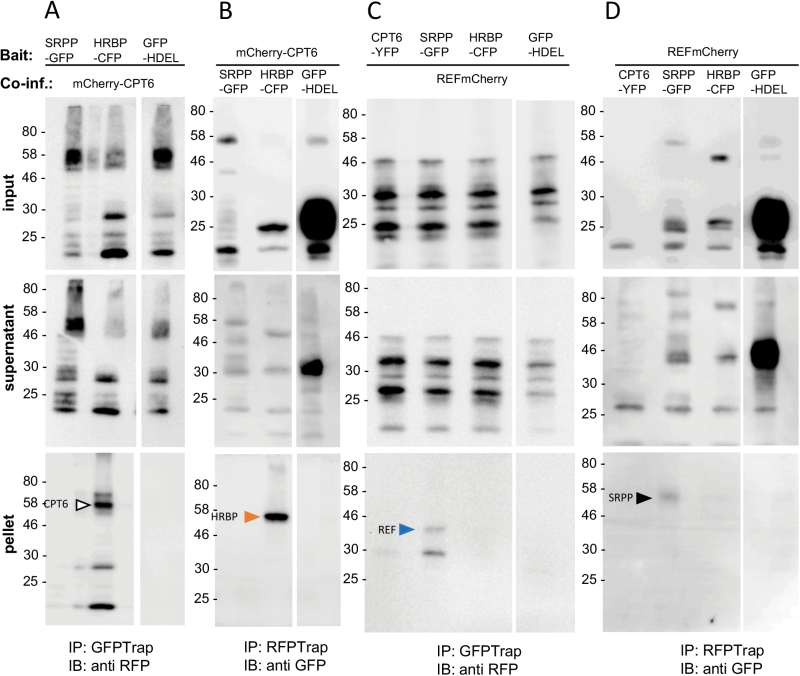
Co-immunoprecipitation of rubber particle-associated proteins. *N. benthamiana* leaves were agroinfiltrated with the indicated combinations of constructs. After 3 d, leaf sectors were homogenized and the bait proteins subjected to immunoprecipitation (IP) with the indicated antibodies. Immunoselected polypeptides were resolved by SDS-PAGE and gels immunoblotted (IB) with the indicated antibodies. Input: total protein extract; supernatant: unbound proteins from the immunoprecipitation step; pellet: immunoprecipitated proteins. The numbers on the left of the blots indicate the position of molecular mass markers (kDa).

In order to validate the protein–protein interactions we observed so far by light microscopy, we performed a series of immunoprecipitation experiments (Fig. 7). Interactions were tested between RP proteins fused to GFP or its relatives CFP, eGFP and YFP, *versus* those with RFP-based fluorescent protein fusions, which included RFP and mCherry. Given that the position of the fluorescent protein tag did not seem to affect the localization of CPT6, REF and SRPP, we only used constructs where the tag was fused to the C-terminus of each protein for our co-immunoprecipitation experiments. Constructs were co-infiltrated into *N. benthamiana* leaf epidermal cells. The tissue was harvested and rubber particle protein–fluorescent protein fusions were immunoprecipitated with nanobodies bound to agarose beads. CFP, GFP and YFP were immunoprecipitated with an anti-GFP nanobody, while RFP and mCherry were immunoselected with an anti-RFP nanobody. We then detected the presence of any interacting partners by immunoblot with anti-RFP or anti-GFP antibodies specific to the tag of the putative interactor. The assay was performed in both directions, i.e. using each of the putative interacting proteins in turn as the bait. The expected size of each protein was determined by immunoblots conducted on total extracts of infiltrated leaves (see Supplementary Fig. S5). Figure 7A, B shows that HRBP-CFP clearly interacts with mCherry-CPT6. We were not able to co-immunoprecipitate SRPP-GFP and mCherry-CPT6, although co-expression of GFP-SRPP+CPT6-YFP seems to be sufficient to redirect CPT6 to the ER (Fig. 2). However, we detected interaction between SRPP-GFP and REF-mCherry (Fig. 7B, C). No interaction was detected between CPT6 and REF. We also could not detect interaction between HRBP and REF; however, Yamashita *et al.* (2016) were able to detect that interaction by both immunoprecipitation and yeast two-hybrid screening.

**Fig. 8.**
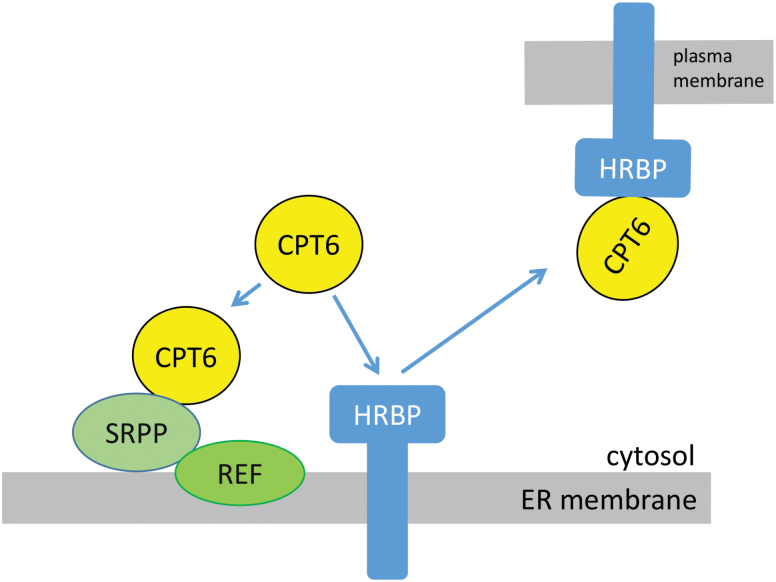
Model of subcellular interactions and localization of rubber particle proteins. Based on our localization and immunoprecipitation data, CPT6 is a cytosolic protein but can be recruited to the ER by co-expression of SRPP. SRPP and REF are both associated with the ER membrane and interact with each other, but the association of SRPP with the ER is likely to be weaker. HRBP is an ER membrane protein. Its interaction with CPT6 leads to both proteins relocating from the ER to the plasma membrane.

Based on our observations, we can conclude that CPT6, while a cytosolic protein, can be recruited to the ER membrane by SRPP (Fig. 8). SRPP interacts with REF, which also co-localizes to the ER membrane. In addition, CPT6 can interact with HRBP but the complex does not stay on the ER membrane but moves to the plasma membrane. This intriguing result mirrors the data obtained by Yamashita *et al.* (2016). It likely indicates that other proteins are required to maintain the CPT6–HRBP complex at the ER. Based on the fact that deletion of TM2 of HRBP leads to the complex remaining in the ER, it is tempting to speculate that TM2 may be part of the site for interaction with a third partner or a wider protein complex.

We have provided an initial localization and established some of the interactions between the most abundant proteins found in the RP and the newly described HRBP. Assuming that the ER membrane of *N. benthamiana* is a reliable model for the ER membrane of laticifer cells in *Hevea*, our data complement those by Yamashita *et al.* (2016) and also highlight some differences. For example, in our experimental system, CPT6 is a stable, easily detected protein (Fig. 7 and Supplementary Fig. S5), while it was never seen when expressed on its own by Yamashita *et al.* (2016). This led the authors to suggest a possible stabilizing role of HRBP, as it was only when HRBP was co-expressed that CPT6 became visible (Yamashita *et al.*, 2016). Here we show that CPT6 is stable in the cytosol, but when either SRPP or HRBP is present the subcellular fate of CPT changes to ER and PM, respectively. Furthermore, we observed that co-expression of SRPP and HRBP displaces CPT6 from the cytosol but does not lead to a significant increase in the fluorescence signal, indicating that the protein is unlikely to be stabilized by this interaction. SRPP, in itself an ER-associated protein, seems to be sufficient to recruit CPT6 to the ER membrane although we have not been able to detect a binary interaction via co-immunoprecipitation. This may be explained by suggesting that the type of interaction between CPT and SRPP is of a weaker nature than that displayed with HRBP, and therefore potentially disrupted during protein extraction. The nature of the association of HRBP with the membrane, and indeed its actual transmembrane topology, remain to be elucidated. We found here that two regions predicted as putative transmembrane domains play different roles for ER location, interaction and recruitment of CPT to the ER and the ability of the CPT–HRBP complex to travel out of the ER and to the PM as its final destination. In the future, it will be important to establish the exact topology of HRBP, in order to establish whether HRBP is indeed a key nucleation factor for a larger complex. Finally, we propose a model that summarizes RP protein interaction at the ER and how HRBP plays a central part in the recruitment of the necessary protein components for the formation of rubber particles (Fig. 8). Taken together, these results support the hypothesis that the rubber transferase complex assembles at the ER membrane and that the *cis*-prenyltransferase CPT6, while a cytosolic protein, can be recruited to this organelle, therefore supporting the idea that rubber particles may originate from the ER membrane.

## Supplementary data

Supplementary data are available at *JXB* online.

Fig. S1. The cytosolic localization of CPT6 is independent of the position of the YFP tag.

Fig. S2. Individual and combined expression of YFP-CPT6 and REF-mCherry.

Fig. S3. Individual and combined expression of GFP-SRPP and YFP-CPT6.

Fig. S4. TOPCONS topology predictions for HRBP and its orthologues NgBR, TbRTA and LEW1.

Fig. S5. Immunoblots of single rubber particle proteins expressed in *N. benthamiana*.

Table S1. Primers used in this study.

Table S2. Constructs generated in this study.

## Supplementary Material

Supplementary_Figures_S1_S5_Tables_S1_S2
