# Supplementary material for: Subcellular localization and interactions among rubber particle proteins from Hevea brasiliensis
Source: J Exp Bot. 2017 Sep 25;68(18):5045–55. doi: 10.1093/jxb/erx331 (PMC5853894; doi:10.1093/jxb/erx331)
Supplement: Supplementary_Figures_S1_S5_Tables_S1_S2 [file erx331_suppl_supplementary_figures_s1_s5_tables_s1_s2.pdf]

Subcellular localisation and interactions among rubber particle proteins from *Hevea brasiliensis*

Daniel Brown[1], Mistianne Feeney[1], Mathin Hamadi[2], Chiara Lonoce[1,$], Roslinda Sajari[3], Alessandra Di Cola[2,€], Lorenzo Frigerio[1,€]

SUPPLEMENTARY DATA

Supplementary Figure S1. The cytosolic localization of CPT6 is independent of the position of the YFP tag
N. benthamiana leaves were agroinfiltrated with the indicated constructs and leaf sectors were imaged after 3 days. Scale bars: 20 µm

Supplementary Figure S2. Individual and combined expression of YFP-CPT6 and REF-mCherry
N. benthamiana leaves were agroinfiltrated with the indicated constructs and leaf sectors were imaged after 3 days. A-C: expression of YFP-CPT6 alone does not result in detectable signal in the mCherry channel. D-F expression of REF-mCherry alone does not result in detectable signal in the YFP channel. G-I: YFP-CPT6 and REF-mCherry do not colocaise, with REF labelling the nuclear envelope and CPT6 labelling the nucleoplasm. Scale bars: 10 µm (A-C, D-F) and 5 µm (G-I).

Supplementary Figure S3. Individual and combined expression of GFP-SRPP and YFP-CPT6
N. benthamiana leaves were agroinfiltrated with the indicated constructs and leaf sectors were imaged after 3 days. A-C: expression of YFP-CPT6 alone does not result in detectable signal in the GFP channel. D-F expression of GFP-SRPP alone does not result in detectable signal in the YFP channel. G-I: YFP-CPT6 and GFP-SRPP both label the nuclear envelope, indicating colocalisation in the endoplasmic reticulum. Scale bars: 10 µm.

Supplementary Figure S4. TOPCONS topology predictions for HRBP and its orthologues NgBR, TbRTA and LEW1.

Supplementary Figure S5. **Immunoblots of single rubber particle proteins expressed in N. benthamiana. A:** RFP fusions, detected with anti RFP. **B:** GFP fusions, detected with anti GFP.

Supplementary Table 1. Primers used in this study.

Supplementary Table 2. Constructs generated in this study.

Supplementary Fig. S1

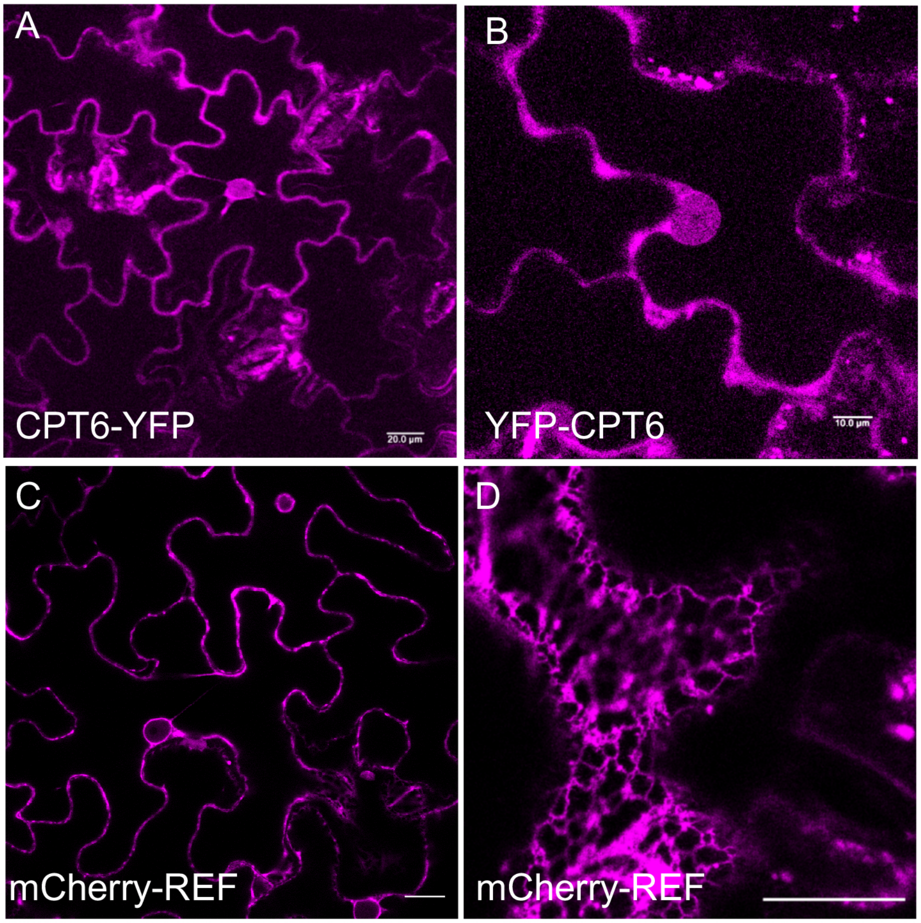

Supplementary Fig. S2

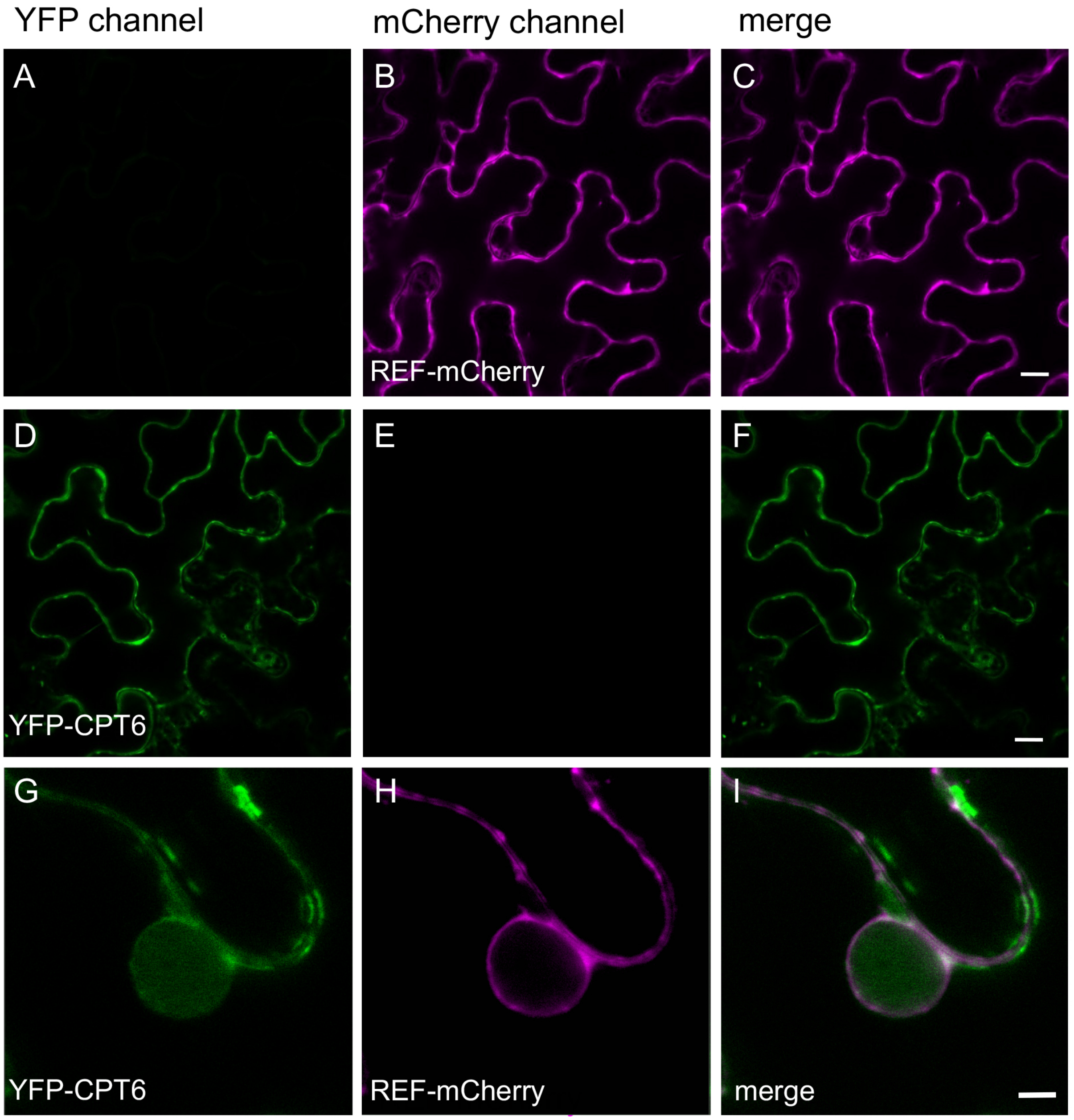

Supplementary Fig. S3

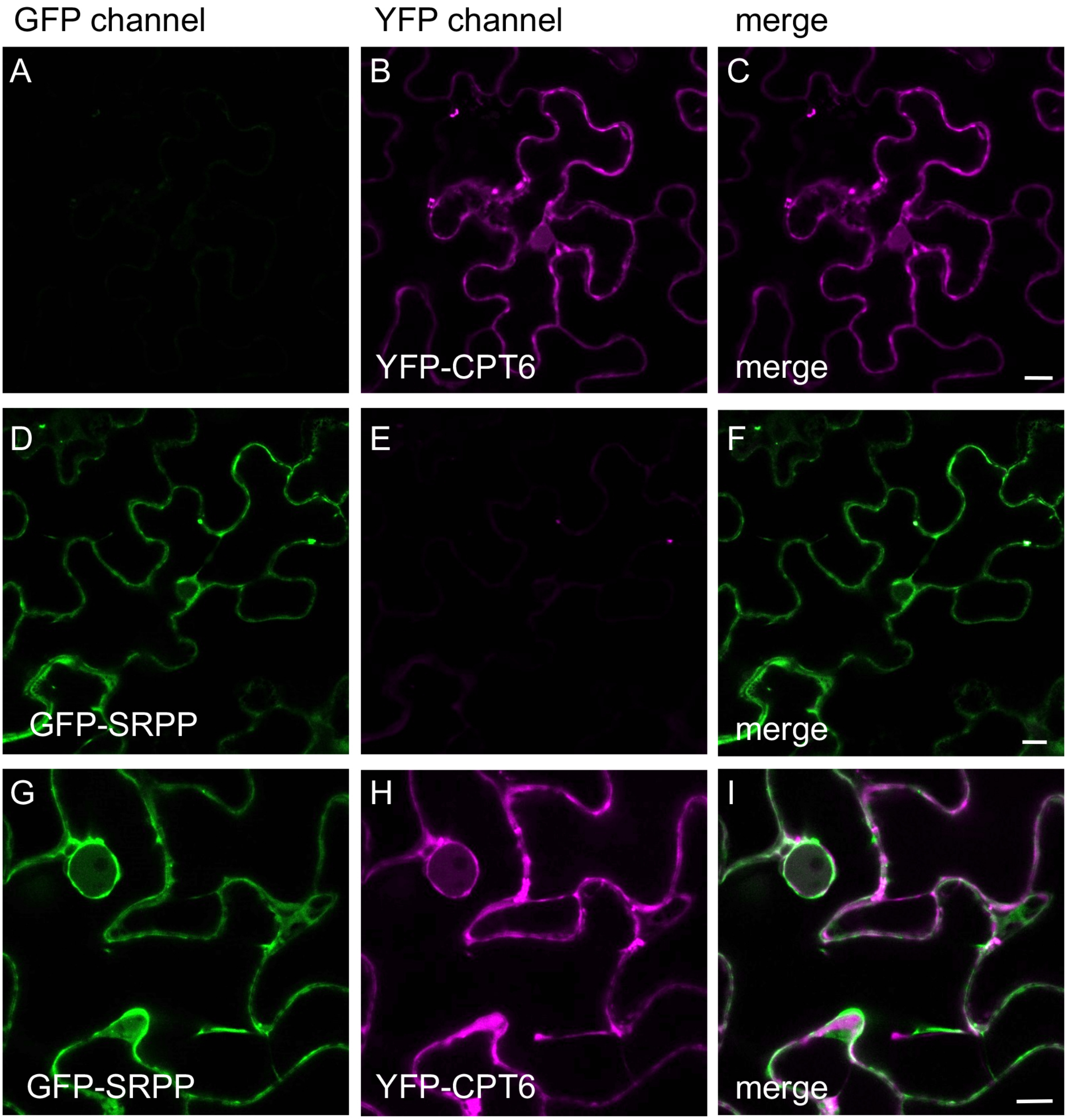

Supplementary Fig. S4

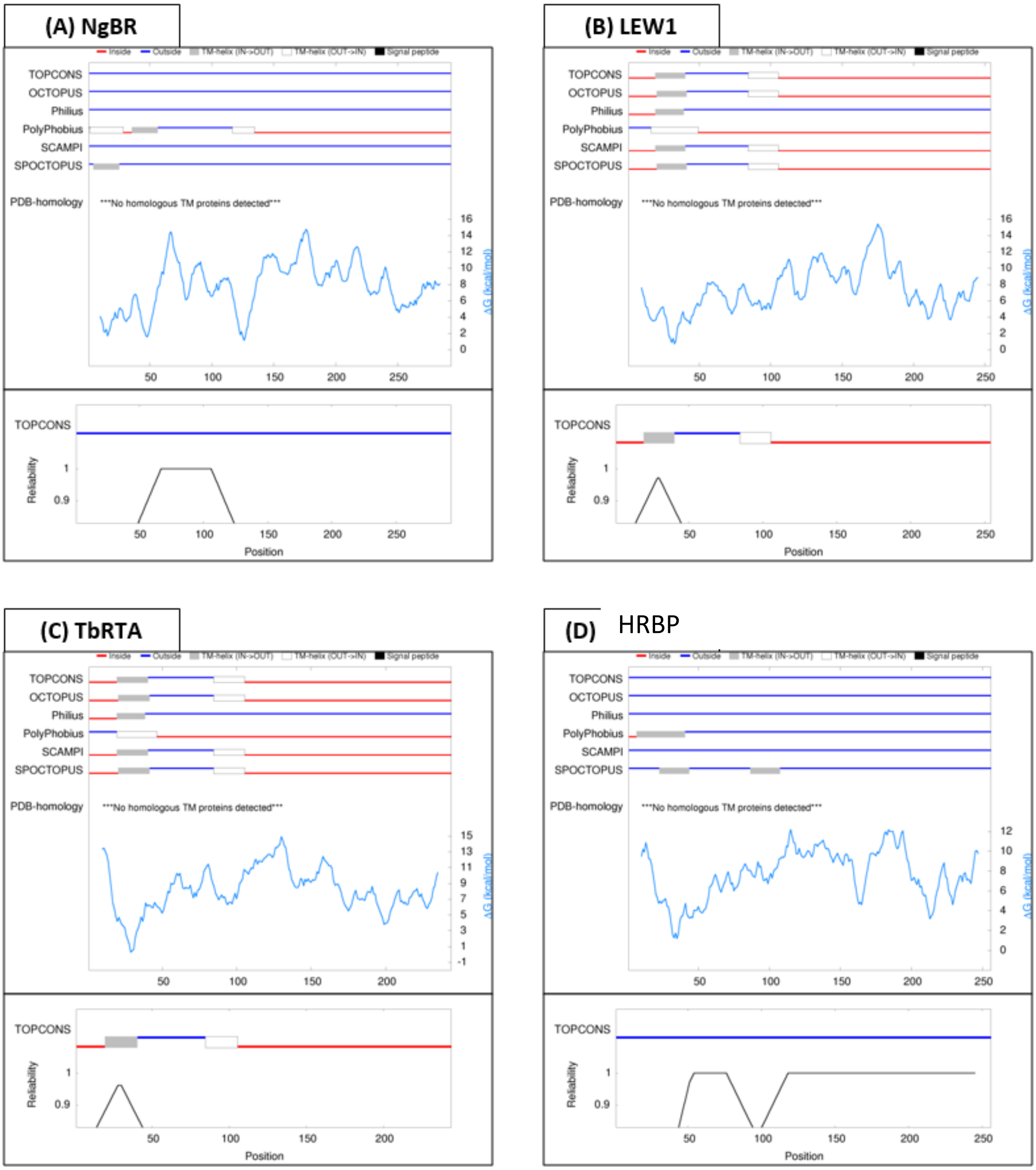

Supplementary Fig. S5

A

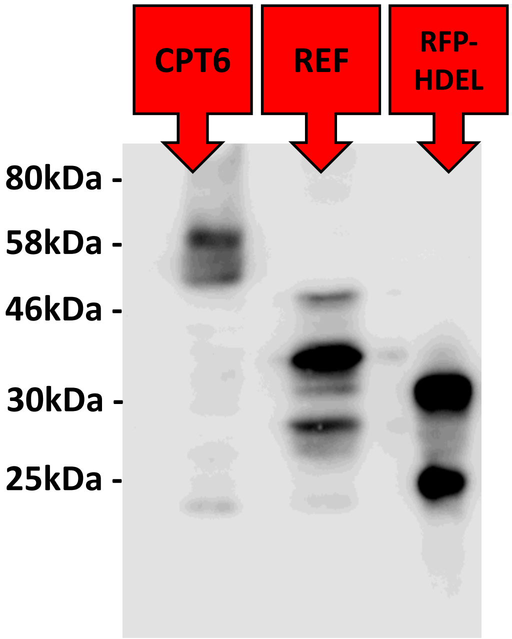

B

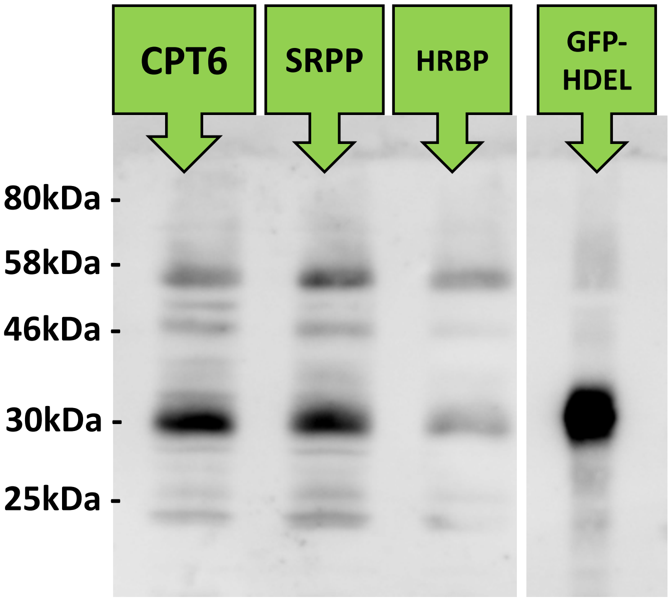

Supplementary Table 1

List of oligonucleotides used for cloning

Construct	Primer	Sequence 5' - 3'
P	*Xba*I FWD	CTATAGCTAGACCTAAAATCATGACC
	CPT6…YFP RVS	CTCCTCGCCCTTGCTGCCCATTTTTAAGTATTCCTTATGTTTC
	CPT6…YFP FWD	GAAACATAAGGAATACTTAAAAATGGGCAGCAAGGGCGAGGAG
	*Sac*I RVS	CTATAGCTAGACCTAAAATCATGACC
35S:YFP-CPT6	*Xba*I FWD	CAGATATCTAGAATGGGCAGCAAGGGCGAGGAGC
	YFP…CPT6 RVS	CTCACCGTTGTATAATTCCATGATCACCTTGTACAGCTCGTC
	YFP…CPT6 FWD	GACGAGCTGTACAAGGTGATCATGGAATTATACAACGGTGAG
	*Sac*I RVS	CAGATATCTAGAATGGGCAGCAAGGGCGAGGAGC
35S:CPT6-mCherry	*Xba*I FWD	CTATAGCTAGACCTAAAATCATGACC
	CPT6…mCherry RVS	GTCCTCCTCGCCCTTGCTCACCATCTCCTCGCCCTTGCTGCCCAT
	CPT6…mCherry FWD	GAAACATAAGGAATACTTAAAAATGGTGAGCAAGGGCGAGGAGGAC
	*Sac*I RVS	CAGATATCTAGAATGGGCAGCAAGGGCGAGGAGC
35S:mCherry-CPT6	*Xba*I FWD	CAGATATCTAGAATGGTGAGCAAGGGCGAGGAGGAC
	mCherry…CPT6 RVS	CTCACCGTTGTATAATTCCATCTTGTACAGCTCGTCCATGCCGC
	mCherry…CPT6 FWD	GCGGCATGGACGAGCTGTACAAGATGGAATTATACAACGGTGAG
	*Sac*I RVS	CAGATAGGATCCTTACTTGTACAGCTCGTCCATGCCG
35S:SRPP-GFP	*Xba*I FWD	CAGATATCTAGAATGGCCGAAGGCGA
	SRPP...GFP RVS	CTCCTCGCCCTTGCTCACCATGCTGGAAACAAGTGGCATG
	SRPP...GFP FWD	CAATCCATGCCACTTGTTTCCAGCATGGTGAGCAAGGGCGAGGAGCTGTTC
	*Sac*I RVS	CAGATAGAGCTCTTACTTGTACAGCTCGTCCATGC
35S:GFP-SRPP	*Xba*I FWD	CAGATATCTAGAATGGTGAGCAAGGGCGAGGAGCTG
	GFP…SRPP RVS	CGTTTCCTTCGCCTTCGGCCATCTTGTACAGCTCGTCCATGCCGAG

	GFP…SRPP FWD	CTCGGCATGGACGAGCTGTACAAGATGGCCGAAGGCGAAGGAAACG
	*Sac*I RVS	CAGATAGAGCTCTCAGCTGGAAACAA
35S:REF-mCherry	*Xba*I FWD	CAGATATCTAGAATGGCTGAAGACGAAGACAACCAAC
	REF…mCherry RVS	GTCCTCCTCGCCCTTGCTCACCATATTCTCTCCATAAAACACCTTAG
	REF…mCherry FWD	CTAAGGTGTTTTATGGAGAGAATATGGTGAGCAAGGGCGAGGAGGAC
	*Bam*HI *RVS*	CAGATAGGATCCTTACTTGTACAGCTCGTCCATGCCG
35S:mCherry-REF	*Xba*I FWD	CAGATATCTAGAATGGTGAGCAAGGGCGAGGAGGAC
	mCherry…REF RVS	GGTTGTCTTCGTCTTCAGCCATCTTGTACAGCTCGTCCATGCCGC
	mCherry…REF FWD	GCGGCATGGACGAGCTGTACAAGATGGCTGAAGACGAAGACAACC
	*Bam*HI *RVS*	CAGATAGGATCCTCAATTCTCTCCATAAAACACCTTAG
35S:HRBP-CFP	*Xba*I FWD	CAGATATCTAGAATGGATTTGAAACCTGGAG
	HRBP…CFP RVS	CTCCTCGCCCTTGCTCACCATCTAACCATAATTTTGCTGCAC
	HRBP…CFP FWD	GTGCAGCAAAATTATGGTTAGATGGTGAGCAAGGGCGAGGAG
	*Sac*I RVS	CAGATAGAGCTCCTAACCATAATTTTGCTGCAC
35S:CFP-HRBP	*Xba*I *FWD*	CAGATATCTAGAATGGTGAGCAAGGGCGAG
	CFP…HRBP RVS	CTCCAGGTTTCAAATCCATCTTGTACAGCTCGTCCATGCCG
	CFP…HRBP FWD	CGGCATGGACGAGCTGTACAAGATGGATTTGAAACCTGGAG
	*Sac*I RVS	CAGATAGAGCTCTTACTTGTACAGCTCGTCC
35S:HRBPΔTM1-CFP	ΔTM1 FWD	AAACGCTATGGAGCCCTC
	ΔTM1 RVS	ATGTAGAGTACGCCACAG
35S:HRBPΔTM2-CFP	ΔTM2 RVS	GGAGTTCTCAAGACAAACAAG
	ΔTM2 FWD	AACTTTAGAAATTTGGTAAGCTTC
CPT6 Gateway Entry	attb1 FWD	AAAAAAGCAGGCTTCATGGAATTATACAACGGTGAGAG
	attb2 RVS	CAAGAAAGCTGGGTCTTTTAAGTATTCCTTATGTTTC
	attb2 RVS (no stop codon)	CAAGAAAGCTGGGTCTCATTTTAAGTATTCCTTATGTTTC

SRPP Gateway Entry	attb1 FWD	AAAAAAGCAGGCTTCATGGCCGAAGGCG
	attb2 RVS	CAAGAAAGCTGGGTCTCAGCTGGAAACAAG
	attb2 RVS (no stop codon)	CAAGAAAGCTGGGTCGCTGGAAACAAG
REF Gateway Entry	attb1 FWD	AAAAAAGCAGGCTTCATGGCTGAAGACGAAGACAACCAAC
	attb2 RVS	CAAGAAAGCTGGGTCTTACTTGTACAGCTCGTCCATGCCG
	attb2 RVS (no stop codon)	CAAGAAAGCTGGGTCCTTGTACAGCTCGTCCATGCCG
HRBP Gateway Entry	attb1 FWD	AAAAAAGCAGGCTTCATGGATTTGAAACCTG
	attb2 RVS	CAAGAAAGCTGGGTCTCATGTACCATAATTTTG
	attb2 RVS (no stop codon)	CAAGAAAGCTGGGTCTGTACCATAATTTTG
Gateway att (secondary) primers	FWD	GGGGACAAGTTTGTACAAAAAAGCAGGCT
	RVS	GGGGACCACTTTGTACAAGAAAGCTGGGT

Supplementary Table 2

List of constructs generated during this work

**Construct**	**Vector**	**Description**
35S:CPT6-YFP	pGreenII-0029	Full length genomic DNA of HRT2 fused in frame with a YFP tag at the C-terminus
35S:YFP-CPT6	pGreenII-0029	Full length genomic DNA of HRT fused in frame with a YFP tag at the N-terminus
35S:CPT6-mCherry	pGreenII-0029	Full length genomic DNA of HRT2 fused in frame with an mCherry tag at the C-terminus
35S:mCherry-CPT6	pGreenII-0029	Full length genomic DNA of HRT2 fused in frame with an mCherry tag at the N-terminus
35S:SRPP-GFP	pGreenII-0029	Full length cDNA of SRPP fused in frame with an GFP tag at the C-terminus
35S:GFP-SRPP	pGreenII-0029	Full length cDNA of SRPP fused in frame with an GFP tag at the N-terminus
35S:REF-mCherry	pGreenII-0029	Full length cDNA of REF fused in frame with an mCherry tag at the C-terminus
35S:mCherry-REF	pGreenII-0029	Full length cDNA of REF fused in frame with an mCherry tag at the N-terminus
35S:HRBP	pGreenII-0029	Full length cDNA of HRBP
35S:HRBP-CFP	pGreenII-0029	Full length cDNA of HRBP fused in frame with a CFP tag at the C-terminus
35S:CFP-HRBP	pGreenII-0029	Full length cDNA of HRBP fused in frame with a CFP tag at the N-terminus
35S:HRBPΔTM1-CFP	pGreenII-0029	A 35S:HRBP-CFP mutant lacking residues 32-58

35S:HRBPΔTM2-CFP	pGreenII-0029	A 35S:HRBP-CFP mutant lacking residues 87-108
35S:HRBP-RFP	pGWB654	Full length cDNA of HRBP with an RFP tag in frame with the c’ terminus
